# Useful parameters for the motion analysis of facial skin care in Japanese women

**DOI:** 10.1186/s40101-020-00234-w

**Published:** 2020-08-24

**Authors:** Shingo Sakai, Ruako Takatori, Mika Nomura, Kuniaki Uehara

**Affiliations:** 1 Skincare Products Research, Kao Corporation, 5-3-28, Kotobuki-cho, Odawara, Kanagawa 250-0002 Japan; 2 Graduate School of System Informatics, Kobe University, 1-1, Rokkodai, Nada, 657-8501 Japan; 3 Present address: Core Device Development Sec., Service Development HQ, R&D Dept., Dwango Co., Ltd., Kabukiza Tower, 4-12-14 Ginza, Chuo-ku, Tokyo, 104-0061 Japan; 4 Kansei Value Development Sensory Science Research, Kao Corporation, 5-3-28, Kotobuki-cho, Odawara, Kanagawa 250-0002 Japan

**Keywords:** Facial skin care, Motion capture, Autonomic nerve system, Relaxation, Approximate entropy, Power law scaling exponent

## Abstract

**Background:**

Facial skin care (FSC) is an important routine for Japanese women. Hand motions during FSC physically affect psychological state. However, it is very difficult to evaluate hand motions during personal and complex FSC. The objective of this study was to find out objective and quantitative parameters for hand motions during facial skin care (FSC). Women who enjoy and soothe during FSC (Enjoyment group (E group), *n* = 20) or not (non-enjoyment group (NE group), *n* = 19) were recruited by an advance questionnaire. The same lotion, emulsion, and cream were provided to all subjects, and they used sequentially in the same way as the women’s daily FSC. The motion of the marker on the back side of the right middle finger during FSC was tracked by a motion capture system. The heart rate variability (HRV) was also measured before and after FSC for evaluating psychological effect.

**Results:**

The averaged acceleration (Avg. ACC), approximate entropy (ApEn), and power law scaling exponent (Rest γ) of the cumulative duration of slow motion from the sequential data of acceleration were evaluated. Compared to the NE group, the E group showed a lower Avg. ACC when using emulsion (*p* = 0.005) and cream (*p* = 0.007), a lower ApEn when using emulsion (*p* = 0.003), and a lower Rest γ (*p* = 0.024) when using all items, suggesting that compared to the NE group, the E group had more tender and regular motion, and sustainable slow motions, especially in the use of emulsion. In the E group, the low/high-frequency component of HRV decreased significantly after FSC, suggesting suppression of sympathetic activity (*p* = 0.045). NE group did not. For all subjects, ApEn and Rest γ showed significantly positive correlation with the increase in the low/high-frequency component of HRV after FSC (*p* < 0.01). ApEn showed significantly negative correlation with the increase in the high-frequency component of HRV after FSC (*p* < 0.05). Avg. ACC did not show significant correlation with them. These results suggested that the behavior of FSC influences the autonomic nerve system.

**Conclusions:**

ApEn and Rest γ are useful parameters for evaluating quality of hand motions during FSC.

## Background

Facial skin care (FSC) is important in maintaining and improving skin function. Ingredients of FSC products are known to improve skin problems such as wrinkles, spots, and rough skin texture [1, 2]. Furthermore, the use of certain hand motions during FSC is expected to have beneficial physiological effects. FSC of Japanese woman has also been reported to influence autonomic nerve system (ANS) activity [3–7]. Alternatively, facial massage by hand is thought to provide physical stimulation to both skin and muscle, leading to morphological and physical improvement [8, 9].

Moreover, FSC combines not only cultural aspects for beauty but also the sociological significance based on communication. It is important to expand the psychological and physiological potential of user by providing the method of FSC.

However, since FSC behavior is very personal and complicated, objective and quantitative evaluation is difficult. There are very few reports on the relationship between hand motions during FSC and physiological changes.

We focused on two parameters, the approximate entropy (ApEn) [10] used for the analysis of complex systems and Rest γ [11] representing the cumulative frequency distribution of human-activity duration for the evaluation of FSC motions. ApEn can be used to evaluate the complexity of information, even for discrete data with finite length [10, 12]. ApEn is smaller in the case of more regular sequenced data. Pincus et al. reported on the risk of sudden infant death syndrome by ApEn using electrocardiogram data [13], while Arif et al. evaluated the change in the stability of walking with aging by ApEn [14]. On the other hand, Nakamura et al. proposed Rest γ as an appropriate living activity index [11]; they binarized sequenced acceleration data, recorded by a locomotor, with the averaged value; from this, the logarithm of the cumulative duration and frequency from sequenced data of the duration of motions smaller than the average value was calculated, and the slope taken as the Rest γ value. Furthermore, they reported that Rest γ indicated qualitative differences in the living activities between healthy and depressed individuals [15].

Many relationships between skills and motion have been reported in the fields of sport [16–18], music [19], and dance [20]. Self-facial massage of Japanese women stimulates the parasympathetic nerve, suggesting a relaxation effect [3, 5–7]. Daily FSC may have the ability to control ANS unconsciously. In these studies, ANS activity is evaluated by heart rate variability (HRV) [21]. HRV, the cardiovascular signal variability of the R–R period, is a commonly adopted marker of cardiac ANS activity, which is both reliable and non-invasive [21]. The frequency of HRV has been analyzed using methods such as fast Fourier transform (an autoregressive model) and maximum entropy methods (MEMs). When the power is separated between high frequency (HF; 0.15–0.45 Hz) and low frequency (LF; 0.04–0.15 Hz) in spectral analysis, the HF reflects the activity of the parasympathetic nervous system and the LF reflects the activities of both the sympathetic and parasympathetic nervous system [22]. The ratio of LF and HF (L/H) can therefore be used as an index of sympathetic nervous system activity [23]. The change of HRV is also closely related to emotional change [24–26].

This study involved two female groups, recruited by advance questionnaire; one group consisted of women who enjoyed and soothed during FSC (E group) and the other group consisted of women who did not enjoy or soothe during FSC (NE group). The FSC behavior of both groups was compared by ApEn and Rest γ. Moreover, we compared the effect of hand motions during FSC on ANS activity change in both groups and the relationship between parameters of FSC motion and ANS activity change.

## Methods

### Subjects

This study included healthy females between 31 and 49 years of age. Subjects who smoked, had skin issues (such as atopic dermatitis), were taking medication, or had received cosmetic surgery were excluded from this study. Subjects used all three items (lotion, emulsion, cream) every day for skin care. Subjects were selected and classed into two groups by the advance questionnaire. The advance questionnaire had two questions: “Do you enjoy during daily FSC?” and “Do you soothe during daily FSC?”. Female answering “Yes” and “No” to both were classed into enjoyment group (E group, *n* = 20; averaged age, 40 ± 4) and non-enjoyment group (NE group, *n* = 19; averaged age, 41 ± 5), respectively. The difference in the age of both groups was not significant (*p* = 0.470). There was no significant difference in subjective skin care time between the two groups (E group, 7.6 ± 3.0 min; NE group, 7.4 ± 3.2 min, *p* = 0.860). All subjects used the same commercially available skincare items (lotion, emulsion, and cream) for FSC in this study.

### Procedure

Subjects were instructed to wear a headband and ring with marker on a chair, at a table, in front of a mirror, for 10 min, and use the lotion, emulsion, and cream sequentially in the same way as their daily FSC. The room was air-conditioned (25 ± 1 °C; humidity, 50% ± 5%), and HRV was measured for 3 min before and after FSC.

The characteristics of items used are shown below.

The lotion:

A typical lotion solubilized with nonionic and silicone surfactants, providing compatible feeling to the skin.

The emulsion:

A typical O/W emulsion combining anionic surfactant and thickener, providing compatible feeling to the skin and elastic feeling.

The cream:

A typical O/W cream with a large amount of water-holding oil, providing soft and fast compatible feeling to the skin. The cream is softer than normal massage one.

### Measurement

**Fig. 1:**
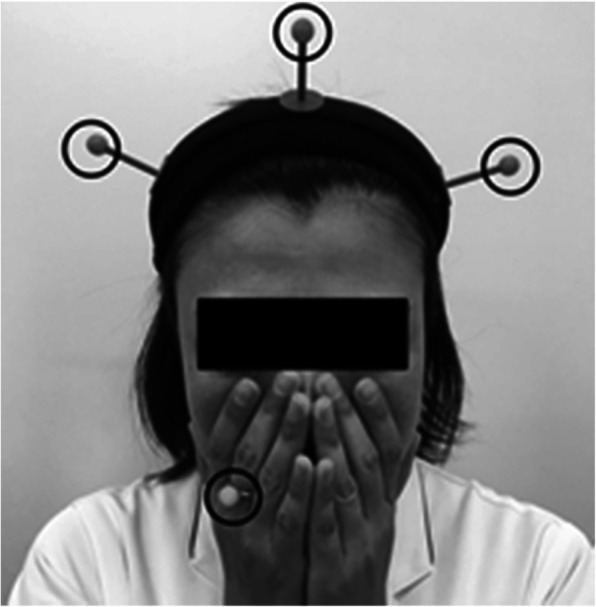
Positions of reflective markers. Circle, position of markers

To measure motion of light hand during FSC, 10-mm reflective markers were affixed to the back side of the base of the right middle finger and on both ends and middle of the head band (Fig. 1). Trajectories were recorded using a 6 camera, three-dimensional motion capture system, sampling at 100 Hz (VENUS3D v.4.1, Nobby Tech Co., Tokyo, Japan).

To measure the HRV of subjects, the left forearm and right ankle of the subjects was fixed by clip-type electrodes (TE-43, Fukuda Denshi Co., Ltd., Tokyo) for electrocardiogram (ECG) monitoring. The HRV was recorded with a Marquette Holter recorder (LRR-03; GMS, Inc., Tokyo, Japan) for 3 min before and after FSC. HRV data were processed using HRV analysis software (Crosswell, Inc., Tokyo, Japan) [27–31].

### Data analysis

The space of the trajectories of the marker on the right middle finger was fixed by a plane and determined by the 3 points on the head band. Sequenced data of acceleration of the marker on the right middle finger was calculated from the corrected trajectory data. ApEn and Rest γ were calculated from the sequenced data of acceleration.

#### Approximate entropy (ApEn)

ApEn is a complexity parameter of information entropy [10, 32]. It can be applied to finite length and discrete data and is calculated by the following formula [32, 33].

A sequenced data: *a* = *a* (1), *a* (2), …, *a* (*n*)

$$ \mathrm{ApEn}\left(\mathrm{m},\mathrm{r}\right)=-\left({\left(n-m\right)}^{-1}\sum \limits_{i=1}^{n-m}\log \frac{C_i^{m+1}(r)}{C_i^m(r)}\right) $$

$$ {C}_i^m(r)=\sum \limits_{j=1}^{n-m+1}{C}_{ij} $$

$$ {C}_{ij}=\left\{\begin{array}{c}1, if\left\Vert x(i)-x(j)\right\Vert \infty \le r\kern0.5em \\ {}0,\kern0.5em otherwise\ \end{array}\right. $$

$$ x(i)=\left[a(i),\dots, a\left(i+m-1\right)\right] $$

*n* = length of data

*m* = length of compared subsequence

*r* = tolerance for noise

In this study, 2, 500, and 0.15 x (standard deviation) were substituted for *n*, *m*, and *r*, respectively, according to the mathematical theory of Pincus [12]. The window of length of compared subsequence (500 points) was calculated and moved from the start to the end, and the averaged ApEn was determined; a smaller ApEn being indicative of a more regular motion.

#### Rest γ

**Fig. 2:**
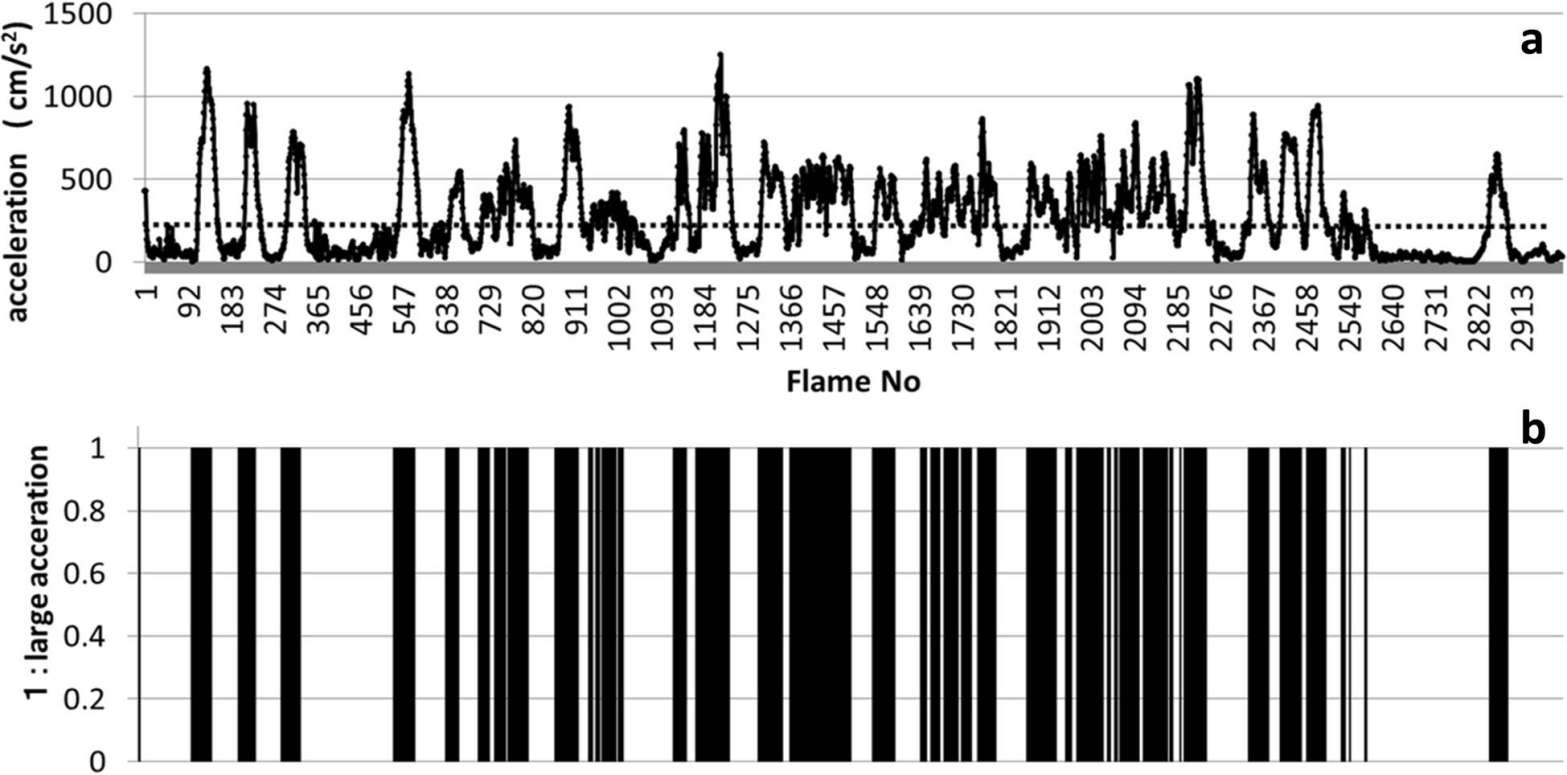
Calculation for Rest ϒ (1). **a** Acceleration series data of marker on right hand is binarized by the averaged acceleration (dashed line). **b** Binarized log of duration of fast motions (black) and slow motions (white) from data **a**

**Fig. 3:**
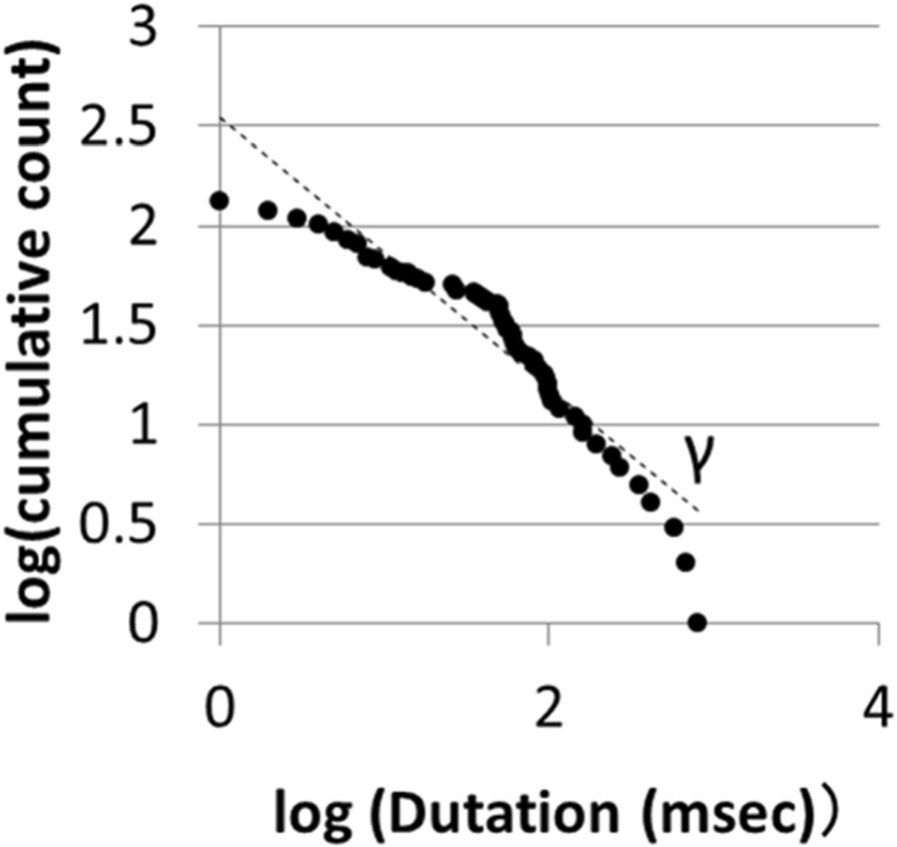
Calculation for Rest ϒ (2). Cumulative distributions of duration of slow motions (white in Fig. 1). The slope (Rest ϒ) is determined by the method of least squares

Using a locomotor, Nakamura et al. demonstrated that the cumulative probability distribution of resting periods in life takes a scale-free power-law form [15]. Rest γ was proposed as a scaling exponent for evaluating quality of life [15, 34, 35]. The sequenced acceleration data was processed according to the method as described by Nakamura et al. [15]. Sequenced data was binarized into “resting periods” or “active periods” by a threshold of averaged acceleration (Fig. 2). Rest γ is a scaling exponent which indicates the cumulative distribution of the frequency for resting periods (Fig. 3). In this study, a lower Rest γ demonstrated a longer sustainability of slow motion or hand pressure.

#### HRV

The power spectrum of a time series of the R-R interval was calculated using the maximum entropy method. The LF and HF power were estimated to be within the 0.04–0.15 and 0.15–0.45 Hz frequency bands, respectively. The LF/HF ratio and HF were taken as parameters of sympathetic and parasympathetic nerve activity, respectively [23]. The coefficient component of variance (ccv) of the LF/HF ratio and the HF were calculated using the following formula [36]:

ccvHF = √HF (ms^2^)/R-R interval (ms)×100

ccv(LF/HF) = √(LF/HF)/R-R interval (ms)×100

### Statistical analysis

All data were analyzed by SPSS (v.25). The effect of time and group was multi compared by the Bonferroni correction method after two-way repeated measures ANOVA. Correlation was analyzed by the Pearson’s simple correlation analysis.

## Results

### Difference in motion parameters between groups

Averaged acceleration (Avg. ACC), ApEn, and Rest γ when using each item (lotion, emulsion, and cream) during FSC in both groups were calculated and compared.

**Fig. 4:**
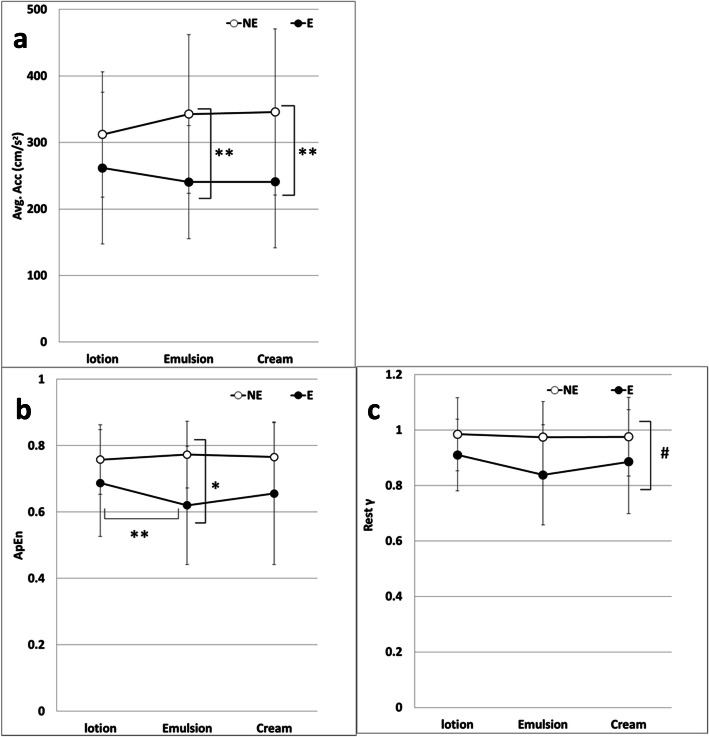
The change of motion parameters during FSC. E, enjoyment group (*n* = 20); NE, non-enjoyment group (*n* = 19). *p < 0.05; **p < 0.01 multiple comparison (Bonferroni correction); ^#^*p* < 0.05 (main effect of group by ANOVA)

With regard to the averaged acceleration, the tendency of interaction between the group and item was analyzed by a two-way repeated measures ANOVA (*F* (1,37) = 2.441, *p* = 0.094). The simple main effect of the item was not significant at the group level (NE group, *F* (2,36) = 1.596, *p* = 0.217; E group, *F* (2,36) = 0.818, *p* = 0.449). The simple main effect of the group was however significant at the level of emulsion and cream (lotion, *F* (1,37) = 2.139, *p* = 0.152; emulsion, *F* (1,37) = 9.127, *p* = 0.005; cream, *F* (1,37) = 8.092, *p* = 0.007). The acceleration of emulsion (*p* = 0.005) and cream (*p* = 0.007) in the E group was significantly smaller than that of the NE group by multivariate ANOVA (Fig. 4a). With regard to the acceleration when using the lotion, the difference between both groups was not significant by multivariate ANOVA (Fig. 4a). These results suggest that the E group has a lower acceleration (gentle motions) than the NE group when using emulsion and cream.

With regard to the ApEn, the relationship between the group and item was also observed by two-way repeated measures ANOVA (*F* (1,37) = 2.612, *p* = 0.080). The simple main effect of items in the E group was significant at the group level (NE group, *F* (2,36) = 0.412, *p* = 0.666; E group, *F* (2,36) = 8.508, *p* = 0.001). The ApEn of the emulsion was significantly smaller than that of lotion (*p* = 0.001) in the E group by multivariate ANOVA (Fig. 4b). The simple main effect of the group was significant at the level of emulsion (lotion, *F* (1,37) = 2.469, *p* = 0.125; emulsion, *F* (1,37) = 10.163, *p* = 0.003; cream, *F* (1,37) = 3.856, *p* = 0.057). The ApEn of emulsion (*p* = 0.003) in the E group was significantly smaller than that of the NE group by multivariate ANOVA (Fig. 4b). These results suggest that the ApEn of emulsion is lower than that of lotion in the E group compared to the NE group.

With regard to the Rest γ, the interaction between the group and item was not significant by two-way repeated measures ANOVA (*F* (1,37) = 0.996, *p* = 0.374). The main effect of the group was significant (*F* (1,37) = 5.522, *p* = 0.024) (Fig. 4c). The tendency of main effect of item was observed (F (1,37) = 3.174, p = 0.054). Multiple comparisons showed that the Rest γ of emulsion was significantly lower than that of lotion (*p* = 0.049). These results suggest that the E group has a lower Rest γ than the NE group.

### Differences in the effects of FSC on ANS activity between groups

With regard to ccv(LF/HF), the interaction between the time and group was significant by two-way repeated measures ANOVA (*F* (1,37) = 4.962, *p* = 0.032). The simple main effect of time in the E group was significant at the group level (NE group, *F* (2,36) = 0.737, *p* = 0.396; E group, *F* (2,36) = 5.339, *p* = 0.027) (Table 1). Multivariate ANOVA showed that the ccv(LF/HF) significantly lowered after FSC in the E group (*p* = 0.013).

**Table 1:**
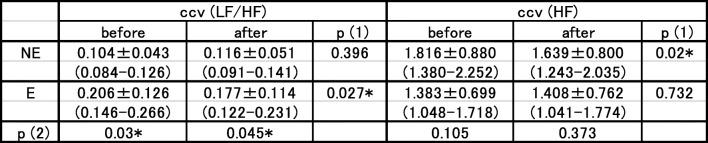
The change of HRV by FSC

NE, non-enjoyment group (*n* = 19); E, enjoyment group (*n* = 20); p (1), before vs after, multiple comparison (Bonferroni correction); p(2), NE vs E, multiple comparison (Bonferroni correction)

*p < 0.05

The simple main effect of the group was significant at the level of time (before, *F* (1,37) = 10.453, *p* = 0.003; after, *F* (1,37) = 4.315, *p* = 0.045) (Table 1). Multivariate ANOVA showed that the ccv(LF/HF) significantly lowered after FSC in the E group (*p* = 0.013). Multivariate ANOVA showed that the ccv(LF/HF) of the E group was higher than that of the NE group before and after FSC. These data showed that the ccv(LF/HF) in the E group was higher than that of the NE group and decreased after FSC.

With regard to ccv(HF), the tendency of the interaction between time and group was analyzed by two-way repeated measures ANOVA (*F* (1,37) = 3.926, *p* = 0.055). The simple main effect of time in the NE group was significant at the group level (NE group, *F* (2,36) = 5.912, *p* = 0.020; *E* group, *F* (2,36) = 0.119, *p* = 0.732) (Table 1). Multivariate ANOVA showed that the ccv(HF) significantly lowered after FSC in the NE group (*p* = 0.020). The simple main effect of the group was not significant at the level of time (before, *F* (1,37) = 2.766, *p* = 0.105; after, *F* (1,37) = 0.812, *p* = 0.373) (Table 1). These data show that the ccv(HF) in the NE group significantly decreases after FSC. These data suggest that effects of FSC on HRV may be different between the NE and E groups.

### The relationship between motion parameters and the change in HRV following FSC

**Fig. 5:**
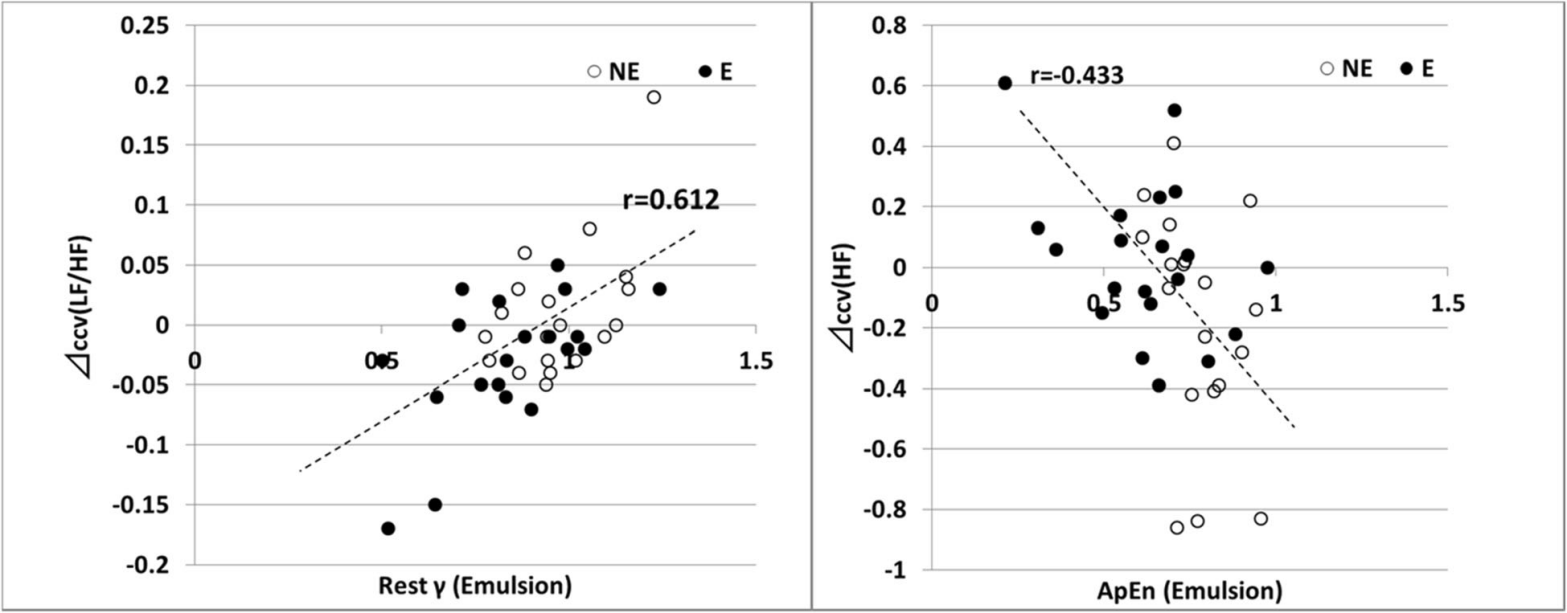
Rest ϒ and ApEn correlates with the change of HRV by FSC (*n* = 39). E, enjoyment group (*n* = 20); NE, non-enjoyment group (*n* = 19)

Both ApEn and Rest γ correlated significantly with the change of ccv(LF/HF) following FSC. Moreover, ApEn significantly correlated with the change of ccv(HF) following FSC (Table 2, Fig. 5). Both parameters showed high correlation especially in the case of emulsion. Averaged accelerator (Avg. ACC) was not correlated with change of HRV. These results suggest that a more regular motion (smaller ApEn) and/or an increased duration of slow motions (lower Rest γ) during FSC may suppress the activity of the sympathetic nerve system, and more regular motion (smaller ApEn) during FSC may stimulate the activity of parasympathetic nerve system independently of the intensity of motion.

**Table 2:**
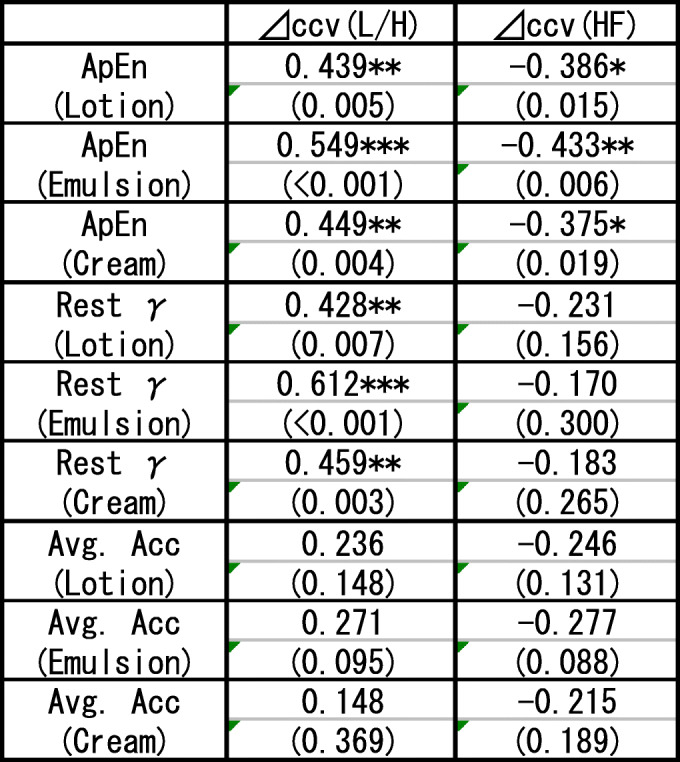
Relationship between motion parameters and changes of HRV by FSC

*n* = 39; ( ), *p* value; *p < 0.05; **p < 0.01; ***p < 0.001

## Discussion

In this study, we have proposed the usefulness of ApEn and Rest γ as quantitative parameters for evaluating FSC behavior. When using the lotion to the emulsion, the E group had a reduced ApEn compared to the NE group. Other two parameters did not show significant change between items. ApEn has been applied in the field of not only neurology [37] and endocrinology [38], but also motion analysis [39]. A lower ApEn indicates a more regular behavior, while higher ApEn values indicate more complex and unpredictable one [32, 40]. In this study, the E group has more regular behaviors than the NE group when using the emulsion. Alkjær et al. demonstrated a higher ApEn of the ankle joint angle when walking in high heels compared to walking barefoot, suggesting a variable adjusting strategy of the nervous system to control the ankle joint [41]. In the context of FSC, differences in the physical property of the emulsion or skin may influence the hand motion; it is certainly known that changes in the physical properties of the object affect the hand motion [42, 43]. However, factors affecting hand motion are more complex due to the context such as purpose [44] and prior knowledge [45]. We must consider further in detail.

Moreover, the E group has a lower Rest γ than the NE group. Rest γ is a universal parameter of a kind of power of law. Nakamura et al. demonstrate the same value for the life activity of healthy humans and mice [15, 34]. The averaged Rest γ value (emulsion) of the E group and NE group is − 0.834 ± 0.181 and − 0.975 ± 0.128, respectively (*p* = 0.012). The Rest γ of the NE group is similar to the value, 1.0, of the life activity of healthy people and mice [34, 35]. It is possible that the motion of the NE group is similar to an unconstrained motion without defect and consciousness. The E group indicates that the duration distribution of the slow motion increases; this may reflect constrained consciousness such as concentration and enjoyment of touching.

Furthermore, we have demonstrated that hand motion during FSC may influence ANS activity. There are some reports demonstrating that FSC provides a relaxing effect as a result of the stimulation of the parasympathetic nerve of the heart [3, 5, 7]. It is interesting that the effects of FSC on ANS activity are different in both groups. It is suggested that FSC of the E group has an inhibitory effect on the sympathetic nerve system (lower ccv(LF/HF)) and that FSC of the NE group has an inhibitory effect on the parasympathetic nerve system (lower ccv (HF)). In this study, the NE group may have trouble carrying out the routine and/or feel stressed when using items during daily FSC. In fact, Okada also suggested that the sympathetic nerve system is stimulated by self-FSC in the case of females with negative attitudes towards FSC [3]. These points require further study.

Interestingly, ApEn and Rest γ during FSC correlated with the change in ANS activity. High ApEn correlated with an increase in sympathetic nerve system activity and a decrease in parasympathetic nerve system activity. These data suggest that more regular (lower ApEn) motion and longer duration of slow motion (lower Rest γ) may lower the balance of ANS activity (sympathetic/parasympathetic nerve system activity). In general, exercise greatly affects the activity of sympathetic nerve system, but it is also known that static stretching activates the parasympathetic nerve system [46, 47]. In this study, the relationship between the acceleration of hand motion and the change of ANS activity is not confirmed, suggesting quality of hand motion but not the intensity of one may affect the balance of ANS activity in the case of FSC. FSC behavior consists of various movement units such as application, tapping, massage, and hand press. In the future, the relationship between each action and ApEn and Rest γ should be examined in detail. It will lead to constitutive understanding of FSC behavior. Feed-back system to user by monitoring ApEn and Rest γ of hand motion during FSC may bring to expand psychological potential for the balance of ANS activity in future.

## Conclusion

ApEn and Rest γ are useful parameters for evaluating quality of hand motions during FSC.

## Data Availability

The datasets during and/or analyzed during the current study are available from the corresponding author on reasonable request.

## Abbreviations

FSC: Facial skin care
E group: Enjoyment group
NE group: Non-enjoyment group
HRV: The heart rate variability
Avg. ACC: The averaged acceleration
ApEn: Approximate entropy
ANS: Autonomic nerve system
HF: High frequency
LF: Low frequency
ANOVA: Analysis of variance
